# Prospects for remodeling the hypertrophic heart with myosin modulators

**DOI:** 10.3389/fcvm.2022.1051564

**Published:** 2022-10-18

**Authors:** Lorenzo R. Sewanan, Yuichi J. Shimada

**Affiliations:** ^1^Department of Medicine, Columbia University Irving Medical Center, New York, NY, United States; ^2^Division of Cardiology, Department of Medicine, Columbia University Irving Medical Center, New York, NY, United States

**Keywords:** hypertrophic cardiomyopathy, remodeling, myosin modulator, mavacamten, hypertrophy, fibrosis, reverse remodeling

## Abstract

Hypertrophic cardiomyopathy (HCM) is a complex but relatively common genetic disease that usually arises from pathogenic variants that disrupt sarcomere function and lead to variable structural, hypertrophic, and fibrotic remodeling of the heart which result in substantial adverse clinical outcomes including arrhythmias, heart failure, and sudden cardiac death. HCM has had few effective treatments with the potential to ameliorate disease progression until the recent advent of inhibitory myosin modulators like mavacamten. Preclinical investigations and clinical trials utilizing this treatment targeted to this specific pathophysiological mechanism of sarcomere hypercontractility in HCM have confirmed that myosin modulators can alter disease expression and attenuate hypertrophic remodeling. Here, we summarize the state of hypertrophic remodeling and consider the arguments for and against salutary HCM disease modification using targeted myosin modulators. Further, we consider critical unanswered questions for future investigative and therapeutic avenues in HCM disease modification. We are at the precipice of a new era in understanding and treating HCM, with the potential to target agents toward modifying disease expression and natural history of this most common inherited disease of the heart.

## Introduction

Hypertrophic cardiomyopathy (HCM) is a structural heart disease historically characterized by left ventricular outflow tract obstruction (LVOTO) and cardiomegaly with severe eccentric hypertrophy (1). At the tissue level, HCM often features cardiomyocyte hypertrophy, myocyte disarray, myofibrillar disarray, interstitial fibrosis, which can result sudden cardiac death, early-onset heart failure with preserved ejection fraction, and end-stage heart disease. Since its discovery, it has come to be recognized as the most common inherited disease of the myocardium which typically results from mutations to the molecular machinery in the cardiac sarcomere (2). Many mechanisms have been proposed that most frequently link mutations to aberrant contractile function and regulation at the level of the sarcomere (3–7).

Conventional agents such as anti-arrhythmics and neurohormonal blocking agents to treat HCM have provided only symptomatic relief though have not modified disease progression. In particular, no therapy has yet been shown to mitigate adverse structural remodeling like hypertrophy and fibrosis. The recently discovered cardiac myosin specific modulator, mavacamten, has been shown in the largest prospective phase 3 trial in HCM to be overall effective in reducing LVOTO gradient and improving objective exercise tolerance [i.e., peak oxygen consumption (pVO_2_)] (8). The efficacy of myosin modulations agents in HCM further raises questions about the potential for reversal or attenuation of adverse structural changes in the hypertrophic heart.

## Pathophysiology and clinical features of HCM

HCM is known to have significant variability in disease course and adverse outcomes in both of its forms, obstructive (oHCM) and non-obstructive (nHCM). In the largest HCM registry to date [SHaRe (Sarcomeric Human Cardiomyopathy Registry)], mortality of younger patients with HCM (age 20–29) was at least 4-fold higher than the general population and 3-fold higher in older patients (age 50–69), indicating that HCM even with contemporary management has death that remains unmitigated and not yet completely preventable (9). Electrical arrhythmias including 20% with atrial fibrillation and 6% with ventricular arrhythmias were quite common as well, with implantable cardioverter-defibrillator (ICD) present in 21% of patients with HCM. Strikingly, 22% of patients developed New York Heart Association (NYHA) III/IV heart failure (HF), especially if diagnosed before the age of 40, and more than 80% of those with HF had left ventricular ejection fraction (LVEF) > 55%. Indeed, HF with preserved ejection fraction and atrial fibrillation were the most common outcomes in HCM patients in this large cohort. A prospective study of 225 patients with nHCM suggested that a 5-year mortality was similar to age-matched and sex-matched general US population (10). However, these patients showed at least a 10% risk of developing NYHA III/IV HF over a median follow-up of 6.5 years. Similar to SHaRe, about 3% of patients developed end-stage heart disease requiring transplantation, even though none had oHCM. Indeed, the progression to left ventricular systolic dysfunction (LVSD) with LVEF < 50% has been documented to occur in 8% of patients with 11% of these patients progressing to cardiac transplant and 2% progressing to left ventricular assist device (LVAD) implantation, a total need for advanced therapy of 13% compared to < 1% of the patients without LVSD (11). The progression of nHCM in HF and arrhythmias does demonstrate overall that HCM structural remodeling of the ventricle and of the atria even without LVOTO remains a significant issue driving disease-related morbidities. Similarly, an HCM imaging registry demonstrating that profound cardiac structural changes with hypertrophy and fibrosis [50% had at least some late gadolinium enhancement (LGE)] in even the milder forms of HCM suggests a need to focus on remodeling as a significant feature driving outcomes of disease from an early stage (12).

The underlying pathophysiology driving HCM cardiac remodeling is complex but must be grounded in an initial understanding of the proximal etiology of HCM. At this time, the mechanisms driving non-sarcomeric HCM remain poorly elucidated, even though sarcomeric mutations than can be linked in a Mendelian fashion to about 40–50% of HCM. However, recent work notes that some HCM may be complex polygenic phenotype with non-sarcomeric disease modifying genes as well as modifiable risk factors such as diastolic blood pressure (13). Lastly, syndromic disease including HCM phenocopies are not at all fully understood but may differ profoundly in their mechanisms (14). Several important observations could be made from a wealth of studies that have accumulated over the last 30 years using biophysical, biochemical, and animal models of disease (15), specifically that HCM-linked sarcomeric mutations tend to increase myofilament calcium sensitivity, increase the crossbridge duty cycle, and increase energy cost of tension generation, leading to a hypercontractile state in cardiac muscle (3–7, 16–36).

Recent translational investigations, for instance, using proteomics and transcriptomics have started to reveal pathways upregulated that could account for hypertrophy and fibrosis in HCM, including ERK, MAPK, AMPK, TGF-β, amongst others (37). Such work further is corroborated by some human iPSC and animal models of disease that show direct linkages between molecular changes caused by genetic changes to the sarcomere and the upregulation of pathways leading to hypertrophy and fibrosis (38–40). These linkages are not fully explained at this time, but they do not necessarily correspond to a typical paradigm of afterload causing hypertrophy and fibrosis as in hypertensive heart disease and valvular heart disease (41, 42). The mechanobiology and mechanisms seem to be somewhat distinctive.

## Myocardial remodeling in HCM

Remodeling in HCM is primarily noted as thickened heart walls which occurs spontaneously and presumably progressively over the life of an individual to the time they present clinically (43). Studies of genotype positive, phenotype negative individuals have demonstrated that the hypertrophy can be subclinically present in many adolescents. A study of 39 children with HCM showed that 22 patients progressed with up to 12 mm wall thickening by 19 years of age (44). However, a similar study of 65 adult patients with HCM demonstrated that continued hypertrophic remodeling rarely occurred in adults (45). Some reverse remodeling of cardiac thickening and even thinning without LVSD does spontaneously occur in some patients though no evidence shows this to have negative clinical impact (43). However, patients can sometimes develop progressive adverse remodeling with LVSD and extensive fibrosis, essentially burnt out heart disease, with poor outcomes (46). Therapies in patients with established HCM therefore would need to address structural remodeling, though it remains to be determined whether reversal of hypertrophy and fibrosis would be more beneficial than preventing hypertrophy and fibrosis at an initial state in the disease course.

Conventional medical therapies for HCM have had limited efficacy in disease modification though have proven useful in particular scenarios with oHCM in reduction of LVOTO and improvement of overall heart function (47). Selective β-blockers are commonly used as they are known to reduce LVOTO gradient with exercise provocation (48). Calcium channel blockers like diltiazem and the sodium channel blocker disopyramide are used for their negative inotrope effect by overall reduction of intracellular calcium, which leads to suppression of sarcomeric activity. Overall, these agents can be effective in reducing LVOTO, controlling symptoms, and even exercise tolerance, though have limited effect structurally. For instance, early administration of diltiazem was not found to be effective in patients with preclinical HCM in preventing progression and development of clinical HCM though only small studies have been conducted at this time. Several agents have been investigated for their potential effect on remodeling with mixed results including perhexiline and trimetazidine (49), ranolazine and eleclazine (50), losartan (51, 52), and spironolactone (53). It appears that many preclinical studies that suggested an effect on HCM through indirect pathways have not panned out in their *in vivo* application, potentially due to a combination of inability of animal models to capture human pathophysiology, differences in HCM pathophysiology across patients and mutations, difficulty in assessing when patients should be treated at an early enough stage to reverse disease, and perhaps a lack of targeting the proximal mechanism of HCM itself.

With regards to evidence for treatment during an early stage of disease, the VANISH trial investigated whether using valsartan for preclinical HCM would have a beneficial effect and was designed on the premise that animal models demonstrate that use of ARBs can inhibit TGF-β dependent remodeling in HCM hearts if treated prior to establishment of disease. With this specifically in mind, the trial enrolled 178 participants with a mean age of 23 and an initial LV wall thickness of 16 mm into a randomized phase 2 clinical trial in which they received either valsartan or placebo for 2 years (54). The endpoint of the study evaluated a complex nine-measure composite endpoint of z score-normalized cardiac magnetic resonance (CMR), echocardiographic, and biomarkers relating to diastolic function, hypertrophy, and myocardial injury. The trial met its endpoint showing that the patients who received valsartan showed less progression in these parameters; specifically, the NT-proBNP and diastolic measures as well as LV wall thickness worsened in the placebo group compared to the treatment group. This demonstrates a therapeutic paradigm of early treatment to prevent HCM complications rather than palliation.

Some of the sickest patients remain those with oHCM, many of whom require progression to septal reduction therapy (SRT) despite medical therapy as no medical therapy has been shown to prevent progression but rather to be temporizing at this time. SRT is indicated when patients have persistent LVOT gradient >50 mmHg and NYHA functional class III/IV or recurrent syncope with maximal medical therapy that can be tolerated (55). In terms of the effect on myocardial structure, it would be hopeful that relief of obstruction and the high afterload state would lead to some degree of remodeling, similar to that of treatment of hypertension, with some improvement in LV systolic function, diastole, and energetics. An early echocardiographic-based retrospective study of 60 patients who underwent septal myectomy showed a reduction of mean LV gradient of 67 mmHg to 12 mmHg, with EF decreasing from 74 to 67% on average, with expected reductions in septal wall thickness and left ventricular end systolic diameter (LVED) (56). Over the course of 2 years, left ventricular end diastolic diameter (LVEDD) was unchanged, but posterior wall thickness decreased mildly by 1 mm and left atrial diameter (LAD) decreased by 3 mm on average. Importantly, LV mass overall decreased from about 300 to 250 g on average, which was maintained at this level for a follow-up of longer than 2 years but was still larger than normal hearts, suggesting that the LVOTO is not the only driver of hypertrophy and fibrosis that leads to cardiomegaly in HCM. A later study of 66 oHCM patients with septal myectomy using echocardiography added measurements of strain (57). This study showed that after myectomy, longitudinal strain decreased at the myectomy site, increased in the lateral segments, but remained unchanged globally, with normalization of ventricular twist.

In order to understand the differences in remodeling after afterload removal in myocardium with intrinsic myocardial abnormality vs. that with presumably normal intact intracellular pathways, it is interesting to consider the structural and functional recovery after myectomy for oHCM and aortic valve replacement (AVR) for aortic valve stenosis, an extrinsic cause of hypertrophy (58). A small prospective study of 10 patients with oHCM and 10 patients with severe aortic stenosis (AS) were examined with echocardiography, CMR, and exercise testing. After AVR, patients experienced decrease on average of mean transvalvular gradient from 49 to 11 mmHg, with decrease in global LV and LA dimensions as well as lateral wall thickening. Global longitudinal strain improved, and exercise capacity improved, with a trend toward improvement in pVO_2_. In oHCM patients with myectomy, LA dimension decreased after myectomy and LV mass/septal thickness as expected, though there was no change in LV dimension specifically. Global longitudinal strain did not improve in oHCM after myectomy, though there was some improvement in exercise capacity but no improvement in pVO_2_. LGE as expected was unchanged after the procedure in either cohort. Comparing these two cohorts, the main comparable effect was that left atrial (LA) volume decreased in both implying improved diastolic function, but oHCM did not recover any strain metrics implying little functional myocardial improvement in the oHCM hearts with remodeling and intrinsic myocardial abnormalities due to the aberrant genetics likely driving that disease.

## Myosin modulator mechanisms and clinical applications

Altogether, it may be that reverse remodeling and improved myocardial function cannot be achieved in HCM by conventional medical therapy or surgical means due to the intrinsic defect of the myocardium itself resulting from the genetic mutation causing HCM. While many pathological mechanisms may be initiated by the various HCM mutations, studies of thick filament mutations in myosin in particular identified hypercontractility and upregulation of crossbridge cycling as a potential drug target (59–62). No suitable agents were available until recently with the discovery mavacamten (MYK-461), a first in class myosin blocker with specificity to cardiac β myosin (63), and a second agent aficamten (64) which is under investigation (NCT04219826).

Biochemical studies demonstrated that mavacamten was able to decrease myosin ATPase activity in a dose-dependent fashion and furthermore decrease maximal tension generation in demembranated cardiac muscle without a change in calcium sensitivity (65). Initially mavacamten was suspected to have an effect on myosin crossbridge cycling by inhibiting release of phosphate from myosin and decreasing the number of actin-binding heads transitioning from weakly to strongly bound state (63) which altogether would decrease force generation. However, ultimately, mavacamten was found to act through a novel mechanism on stabilizing myosin the interacting heads motif (IHM) and locking myosin in the super relaxed state (SRX), thereby completely removing myosin from the cross-bridge cycle itself (66–68). At the tissue level, mavacamten has potent effects on diastole in addition to systole, showing improvement in relaxation, decrease in stiffness, and augmentation of Frank-Starling mechanism in human engineered heart tissue (69). In a seminal study of multiple mouse models of HCM with classic myosin heavy chain (MYH6) mutations (R403Q, R719W, and R453C), mavacamten was shown to decrease fractioning shortening *in vivo* in young and old mice (65). It further prevented hypertrophic remodeling of mouse hearts when given prior to establishment of hypertrophy in young mice. Mechanistically, treated animals also demonstrated normalization of transcriptional pathways that regulate hypertrophy, fibrosis, and energy utilization. However, the effect of reverse remodeling was ameliorated in older mice with established hypertrophy. Altogether, based on this evidence, it was likely that mavacamten could target the pathophysiology of HCM by decreasing myosin availability (31), improving patient outcomes, though it was unclear if this would also have an effect on beneficial cardiac remodeling in the long-term.

Mavacamten was tested in a phase 3 prospective randomized clinical trial (RCT) vs. placebo (8) in which 251 adult patients with symptomatic oHCM were included with LVOTO of >50 mmHg at rest, with Valsalva, or with exercise, preserved LVEF, and NYHA class II-III, with a primary endpoint of improvement in pVO_2_ with at least one NYHA class improvement, or a 3.0 mL/kg per min or greater increase in pVO_2_ with no worsening of NYHA class (EXPLORER-HCM). Primary endpoint was met in 45% of patients compared to 22% of patients on placebo. Importantly, there was a large mean reduction of almost 50 mmHg in post-exercise LVOT gradient which translated to improvement in pVO_2_ of almost 1.4 mL/kg per min on average and further improvement in subjective symptoms as measured by scales such as KCCQ-CSS. Mavacamten had a good safety profile with 97% completion through 30 weeks and no increase in overall adverse events compared to the placebo during the trial. Notably, six patients on mavacamten had transient decrease in LVEF of < 50% not attributed to other causes, though not associated with clinical adverse outcomes. After discontinuation temporarily for three of these patients, the LVEF recovered, and the study was completed. In the other three patients, LVEF was noted to be decreased to around 48% at the end of the study though notably recovered after mavacamten washed out. Therefore, the response of some individuals with lowered EF necessitates long-term monitoring and possible dose adjustments. Longer-term safety profiles are being currently explored (NCT03723655). Furthermore, in a second RCT (VALOR-HCM), in patients who met clinical criteria for SRT and were referred to SRT, there was a 60% reduction in meeting clinical criteria for SRT or proceeding with SRT in those treated with mavacamten compared to placebo at 4 months (70). Mavacamten was approved by the FDA in 2022 for the patient population included in EXPLORER-HCM, specifically patients with oHCM with NYHAII-III with a LVOT gradient >50 mmHg at rest. An initial phase 2 trial (MAVERICK-HCM) has established good safety and tolerability as well as improvement in cardiac biomarkers of mavacamten in patients with nHCM though and long term effects in a randomized clinical trial are pending further study at this time (38).

## Remodeling potential of myosin modulators in obstructive HCM

While effective in relieving symptoms and effect of obstructive HCM through this proximate myosin-targeted mechanism that reduced hypercontractility, LVOTO gradient, and effective afterload on ventricular cardiomyocytes, mavacamten and similar drugs in development may also lead to positive remodeling in human hearts as suggested in animal work. An echocardiographic study on all patients in EXPLORER-HCM (*n* = 251) analyzing changes in key echocardiographic parameters in symptomatic patients with oHCM over 30 weeks recently demonstrated improvements in markers of oHCM (71). There was an increase in LV wall thickness of 1.4 mm in the placebo group over this time period while those treated maintained the same wall thickness. Interestingly, there was resolution of systolic anterior motion (SAM) of the mitral valve in patients with SAM in almost 81% of patients treated with mavacamten. Despite relatively small changes in structure in the echocardiographic study, there was significant and striking effects on LV diastolic function with improvement in septal e′ of 0.7 cm/s, septal E/e′ of −3.5, lateral E/e′ of −3.8, and decrease in left atrial volume index (LAVI) of −7.5 mL/m^2^. A sub-study using CMR imaging explored the effect on structure and function in 35 patients in greater detail (72). The study observed that there was a decrease in LV mass index by median 15.8 g/m^2^, max LV wall thickness by median 2.4 mm, and LA volume index by median 10.3 g/m^2^. At the same time, there was no change in fibrosis markers as evidenced by no significant change in LGE over this time period of 30 weeks though there was little fibrosis in most of the patients at baseline. Interestingly, there was a significant decrease in LVEF by a median 6.4% overall in the HCM group vs. the placebo group of 3.9%; however, none of these had a LVEF < 50% since all began at an elevated baseline of hyperdynamic function. Overall, these findings while early in mavacamten suggest favorable reverse remodeling which correlated with the overall improvement cardiac function in patients treated with mavacamten. LV hypertrophic thinning in patients living out the natural history of their condition is frequently associated with increased collagen replacement and increased myocardial fibrosis. Reassuringly, there was no change in fibrosis as evidenced by LGE seen in this study. Notably, studies of remodeling have not been completed in patients with nHCM and remodeling remains an important and intriguing aspect of the studies as nHCM which can be considered a type of heart failure with preserved ejection fraction (HFpEF) has even more limited treatment options than oHCM.

As seen and hypothesized in prior studies, some of the longer-term changes in cardiac remodeling may require a long study period, though it is encouraging mavacamten has demonstrated favorable changes using a pharmacological therapy that has only been seen in surgical myectomy previously. As seen previously, a profound question of utility of mavacamten in the time course of disease remains to be answered, and it may be that early treatment prior to substantial remodeling may also result in prevention of adverse cardiac remodeling in individuals with HCM though a careful analysis of safety and benefits must be undertaken. Indeed, prior work indicates the majority of HCM remodeling occurs in early adulthood and late teen years, perhaps overall interacting and driven with other hormonal changes in the body at that time that drive overall maturation and growth. It may be possible that targeted treatment during an early period could prevent further HCM changes and may not necessarily necessitate indefinite treatment which would be indeed of utmost desirability for patients. However, such targeting may require further advances in genotype-phenotype associations and early screening programs as it is not altogether feasible at this time to predict which patients even with familial mutations will necessarily develop clinically relevant HCM.

## Conclusions

In this review, we survey mechanisms of novel pharmacological therapies for HCM and their clinical trial evidence and compare their potential for inducing remodeling of the myocardium compared to previous therapies. We find evidence both mechanistically and from clinical trials that induction of reverse remodeling is possible and likely beneficial. While more research is needed, therapies like myosin modulators can induce beneficial cardiac remodeling and possibly prevent further adverse remodeling of hypertrophic hearts. However, important questions about long-term treatment and appropriate time frame specifically earlier therapy remain to be answered.

## Author contributions

LS and YS contributed to the conception, writing, and editing of this manuscript. All authors contributed to the article and approved the submitted version.

## Funding

YS was supported by NIH R01 HL157216, the American Heart Association National Clinical and Population Research Awards, the American Heart Association Career Development Award, Korea Institute of Oriental Medicine, Feldstein Medical Foundation, Columbia University Irving Medical Center Irving Institute for Clinical and Translational Research Precision Medicine Pilot Award, and Columbia University Irving Medical Center Marjorie and Lewis Katz Cardiovascular Research Prize. The funding organizations did not have any role in the study design, collection, analysis, or interpretation of data, in writing of the manuscript, or in the decision to submit the article for publication. The researchers were independent from the funding organizations.

## Conflict of interest

The authors declare that the research was conducted in the absence of any commercial or financial relationships that could be construed as a potential conflict of interest.

## Publisher's note

All claims expressed in this article are solely those of the authors and do not necessarily represent those of their affiliated organizations, or those of the publisher, the editors and the reviewers. Any product that may be evaluated in this article, or claim that may be made by its manufacturer, is not guaranteed or endorsed by the publisher.
